# Supplementary material for: SNPs in lncRNA KCNQ1OT1 Modulate Its Expression and Confer Susceptibility to Salt Sensitivity of Blood Pressure in a Chinese Han Population
Source: Nutrients. 2022 Sep 26;14(19):3990. doi: 10.3390/nu14193990 (PMC9571541; doi:10.3390/nu14193990)
Supplement: Supplementary file 1 [file nutrients-14-03990-s001.zip › nutrients-1895842-supplementary.pdf]

Supplementary Table S1. Demographic and clinical characteristics of participants.

Characteristics	First stage (n=251)	Second stage (n=1443)	All subjects (n=1684)
Gender (male, %)	76(30.3)	371(25.9)	447(26.5) *
Age (years)	63(59-67)	59.29(54-63.90)	59(54-63)
BMI (kg/m^{-2})	25.03(23.11-27.26)	25.92(23.91-28.21)	25.92(23.85-28.20)
WHR	0.90(0.85-0.95)	0.88(0.85-0.93)	0.89(0.85-0.93)
FBG (mmol/L)	5.51(5.01-5.21)	5.44(4.97-6.19)	5.43(4.97-6.17)
TG (mmol/L)	1.60(1.20-2.28)	1.63(1.10-2.53)	1.63(1.12-2.51)
TC (mmol/L)	5.14(4.58-5.70)	4.99(4.27-5.70)	5.01(4.32-5.70)
HDL-C (mmol/L)	1.46(1.25-1.70)	1.45(1.14-2.60)	1.41(1.12-2.40)
LDL-C (mmol/L)	2.71(2.16-3.27)	2.17(1.48-2.87) *	2.25(1.54-2.92) *
Current drinking	147(58.6)	702(49.0) *	781(46.4) *
Current smoking	105(41.8)	206(14.4) *	258(15.3) *
Hypertension (%)	138(55.0)	739(51.6) *	850(50.5) *
BP change 1 (Response to acute salt loading), mm Hg, median (quartile)			
SBP	11.18(2.68-17.68)	9.18(1.18-16.55)	9.43(1.68-16.68)
DBP	-3.10(-6.60-2.00)	-3.60(-7.60-0.40)	-3.60(-7.6-0.90)
MAP	1.33(-3.17-6.49)	0.83(-3.84-5.16)	0.83(-3.84-5.16)
BP change 2 (Response to diuresis shrinkage), mm Hg, median (quartile)			
SBP	-3.50(-12.00-3.50)	-3.50(-10.00-3.00)	-3.5(-10.50-3.00)
DBP	2.50(-2.00-6.50)	2.50(-1.50-6.50)	2.50(-1.50-6.50)
MAP	0.00(-4.33-5.00)	0.50(-3.67-4.83)	0.33(-3.83-4.83)

BMI, body mass index; WHR, waist-hip ratio; FBG, fasting blood-glucose; TG, triglyceride; TC, total cholesterol; HDL-C, high density lipoprotein cholesterol; LDL-C, low density lipoprotein cholesterol; SS, salt sensitivity; SBP, systolic blood pressure; DBP, diastolic blood pressure; MAP, mean arterial pressure.

*, $P<0.05$ for the association with SSBP using correlation method.

Supplementary Table S2. Function annotations of 13 SNPs in 3 SSBP-related lncRNAs.

Gene	SNP	Chr: position	EA/non-EA	GTEX eQTL	CHB MAF	MAF	RegulomeDB Score	HaploReg				3DSNP			
								Promoter histone marks	Enhancer histone marks	DNAse	Motifs changed	Score	Enhancer	Promoter	TFBS
KCNQOT1	rs10832417	11:2631427	T/G	-	0.31	0.30	3a				18 altered motifs	3.82	0	0	1
	rs7925578	11:2708717	G/T	-	0.25	0.29	3a	FAT, GI, MUS	14 tissues	8 tissues	16 altered motifs	15.14	25	7	1
	rs3782064	11:2699927	A/G	-	0.12	0.19	3a	23 tissues		43 tissues	NRSF	200.93	0	108	107
	rs11023840	11:2700338	T/C	1 tissue	0.21	0.15	4					173.87	0	113	65
	rs7103496	11:2708707	T/C	2 tissue	0.16	0.18	4	FAT, GI, MUS	14 tissues	7 tissues	Foxq1,Gcm1,TATA	14.45	25	7	1
	rs58956504	11:2676224	C/T	-	0.07	0.08	3a	23 tissues		39 tissues		15.66	31	0	0
	rs12360708	11:2699697	T/C	-	0.08	0.08	1b	23 tissues		5 tissues	HES1,Hic1,Rad21	144.52	0	104	45
	rs11023582	11:2639138	A/G	1 tissue	0.05	0.05	5				AIRE,FXR	4.26	13	0	0
	rs231354	11:2685121	T/C	1 tissue	0.13	0.13	4		8 tissues		Glis2	2.92	10	0	0
	rs12577654	11:2662099	T/C	-	0.39	0.38	4		MUS	SKIN, LIV	Hmx,SP1	1.51	1	0	1
KCNQ1	rs2411884	11:2714304	C/G	1 tissue	0.25	0.20	5		7 tissues	IPSC	10 altered motifs	2.88	9	0	0
lnc-AGAP1-8:1	rs71402704	2:236562716	G/A	-	0.05	0.03	2b		BLD, GI		4 altered motifs	90.49	4	0	71
lnc-IGSF3-1:1	rs995060	1:116795104	G/C	-	0.26	0.23	3b				CEBPB,Sox,p300	2.73	0	0	0

SNP, single nucleotide polymorphism. Chr, chromosome. non-EA, non-effect allele. CHB, north Han Chinese. MAF minor allele frequency, HWE Hardy-Weinberg equilibrium.

Supplementary Table S3. The association of SSBP-related lncRNA SNP with its expression by eQTL analyses.

Gene	SNPs	Genotype	n=251	ln expression (Mean±SD)	P	P[a]
KCNQOT1	rs10832417	TT	20	-2.030±0.428		
		TG	96	-1.974±0.437	0.020*	0.022*
		GG	135	-1.860±0.410		
KCNQOT1	rs7925578	GG	17	-2.069±0.437		
		GT	91	-1.956±0.434	0.028*	0.021*
		TT	134	-1.871±0.413		
KCNQOT1	rs3782064	AA	10	-2.046±0.452		
		AG	71	-2.003±0.382	0.019*	0.008*
		GG	163	-1.873±0.436		
KCNQOT1	rs11023840	TT	7	-2.068±0.495		
		TC	63	-1.983±0.381	0.045*	0.028*
		CC	178	-1.889±0.435		
KCNQOT1	rs7103496	TT	9	-2.004±0.356		
		TC	73	-2.017±0.381	0.011*	0.009*
		CC	163	-1.868±0.436		
KCNQOT1	rs58956504	CC	5	-1.955±0.304		
		CT	50	-2.023±0.413	1.99e-05*	2.70e-06*
		TT	193	-1.889±0.428		
KCNQOT1	rs12360708	TT	2	-2.072±0.851		
		TC	23	-1.872±0.405	0.833	0.911
		CC	215	-1.921±0.426		
KCNQOT1	rs11023582	AA	0	-		
		AG	20	-2.383±0.413	1.69e-07*	1.50e-07*
		GG	231	-1.87±0.402		
KCNQOT1	rs231354	TT	5	-1.955±0.304		
		TC	52	-2.024±0.412	0.056	0.064
		CC	215	-1.884±0.425		
KCNQOT1	rs12577654	TT	28	-2.122±0.441		
		TC	93	-1.965±0.415	4.54e-04*	4.09-04*
		CC	130	-1.839±0.412		

KCNQ1	rs2411884	CC	9	-2.265±0.270		
		CG	65	-2.068±0.465	4.08e-06*	1.71e-06*
		GG	162	-1.833±0.391		
lnc-AGAP1-8:1	rs71402704	GG	0	-		
		GA	18	-3.195±0.856	0.299	0.331
		AA	233	-2.998±0.766		
lnc-IGSF3-1:1	rs995060	GG	7	-2.459±1.151		
		GC	94	-1.964±0.677	0.215	0.176
		CC	150	-2.091±0.615		

*, P<0.05; [a], adjusted for age and gender.

Supplementary Table S4. Genotype and allele frequencies of lncRNAs SNPs and genotype risks for SSBP.

Gene	SNP	Model	Genotype	n=251	P-value[a]	
					MAP change1	MAP change2
KCNQOT1	rs10832417	Log-Additive	TT vs. TG vs. GG	20/96/135	0.225	0.681
KCNQOT1	rs7925578	Log-Additive	GG vs. GT vs. TT	19/92/141	0.481	0.220
KCNQOT1	rs3782064	Log-Additive	AA vs. AG vs. GG	10/73/168	0.212	0.717
KCNQOT1	rs11023840	Log-Additive	CC vs. CT vs. TT	7/63/181	0.157	0.483
KCNQOT1	rs7103496	Log-Additive	TT vs. TC vs. CC	9/75/167	0.167	0.983
KCNQOT1	rs58956504	Log-Additive	CC vs. CT vs. TT	3/43/205	0.159	0.012*
KCNQOT1	rs11023582	Log-Additive	AA vs. AG vs. GG	0/20/231	0.087	0.210
KCNQOT1	rs2411884	Log-Additive	CC vs. CG vs. GG	10/69/172	0.124	0.605
KCNQOT1	rs12577654	Log-Additive	TT vs. TC vs. CC	28/93/130	0.194	0.846

*, $P<0.05$; P value was calculated by adjusted age, gender, FBG, LDL, smoking, and drinking.
